# Elephant ‘selfies’: Evaluating the effectiveness of Instagram’s warning of the potential negative impacts of photo opportunities with wild animals

**DOI:** 10.1371/journal.pone.0283858

**Published:** 2023-04-06

**Authors:** Lauren A. Harrington, Angie Elwin, Neil D’Cruze

**Affiliations:** 1 Wildlife Conservation Research Unit (WildCRU), Department of Biology, The Recanati-Kaplan Centre, University of Oxford, Tubney, Oxfordshire, United Kingdom; 2 World Animal Protection UK, London, United Kingdom; Cheetah Conservation Fund, Namibia University of Science and Technology, NAMIBIA

## Abstract

Wildlife tourist attractions offering opportunities to observe, touch, and interact with wild animals, are visited by millions of people every year. Wildlife tourism has considerable economic value in many countries and can have positive impacts on wild animal populations (e.g. through habitat protection); it can also have negative impacts on population conservation and individual welfare (due to, e.g. habitat encroachment, disturbance, or disease). The recent phenomenon of ‘wildlife selfies’ shared on social media may seem harmless but can involve animals illegally or unsustainably captured from the wild, kept in poor conditions, or subject to cruel treatment. To address this issue, Instagram introduced a pop-up alert system that is triggered when users search for wild animal selfie hashtags (e.g. #elephantselfie), warning of the potential negative impacts of wildlife selfies on wild animals. Using elephant selfies as a case study, we found that Instagram’s alert was triggered by only 2% of 244 elephant selfie-related hashtags tested. By comparing three pairs of similar hashtags (one of each pair that triggered the warning and one that did not), we were unable to detect a consistent difference in the type of post using each of the hashtags, the popularity of posts, or the sentiment of viewer comments. The warning is not shown when posting an image, or if a post is viewed directly by a follower, only if the post is encountered via a hashtag search. Currently, what is portrayed on social media appears to be inconsistent with apparent recent shifts in social acceptibilty regarding tourism, particularly as concerns direct contact between tourists and elephants. Instagram’s wildlife selfie initiative was commendable but given its apparent lack of effect, we urge Instagram and other social platforms to do more to prevent harmful content from being posted on their platforms and to promote fair, ethical and sustainable interactions between wild animals and people.

## Introduction

More than a billion tourists travel around the world every year (1.5 billion in 2019, [1]). Prior to global travel restrictions due to COVID-19 in 2020 and 2021, the number of international tourists was increasing at an annual rate of between three and seven percent [1]. Many people travel specifically to see wildlife, and for several countries (particularly developing countries) wildlife tourism is the leading foreign exchange earner [2–4]. Whilst some tourists and travellers desire what might be considered a more natural experience and are content to catch a fleeting glimpse of an elusive wild animal from a distance through binoculars, others increasingly demand guaranteed sightings (e.g. [5, 6]), want to be close to wild animals (e.g. [7]), and sometimes even touch them (e.g. [8]). Wildlife tourism can have significant benefits for wild animals (for example, through the support of protected areas and maintenance of important habitats), but there can also be negative impacts associated with human encroachment and disturbance (including, for example, disturbance of feeding and breeding behaviour, and disease transmission) [9–12]. Consequently, there may be a conflict between tourists and tourism operators and the wildlife that the tourists want to see [2].

Driven by peoples’ (tourists) desire to be close to wild nonhuman animals (hereafter animals), wildlife tourist attractions (WTAs) around the world have become hugely popular [13, 14]. WTAs offer tourists and other visitors opportunities to interact with nondomestic animals, in captivity or in the wild. Globally, WTAs offer everything from walking with lions, swimming with dolphins, and feeding crocodiles to visiting sea turtle and civet coffee farms, viewing rescued orangutans, and watching dancing macaques. Like many other aspects of modern human life, these experiences are widely shared on social media (Instagram, Facebook, and others), increasingly as ‘selfies’ (where a person holds out a camera phone and photographs themselves [15, 16]). Here we are concerned with a particular type of selfie–‘wildlife selfies’–in which photographers capture themselves with a wild animal in the same frame. The popularity of wildlife selfies is such that a number of species found in popular tourist destinations are used as part of a photo-prop industry where tourists pay to pose with them for photographic souvenirs. Examples include the common caiman, *Caiman crocodilus crocodilus*, and green anaconda, *Eunectes murinus*, in Brazil, [17], brown-throated three-toed sloth, *Bradypus variegatus*, in Brazil and Peru, [17, 18], slow loris, *Nycticebus* spp., in Thailand, [19], and barbary macaque, *Macaca sylvanus*, in Morocco, [20]. Wildlife selfies are presumably well intentioned by the person taking the photo, and may appear to be harmless, but tourist attractions offering wildlife selfie opportunities can have negative impacts on local populations of the species involved, and on the welfare of the individual animals (c.f. [13]), depending on where the animals are sourced, the conservation status of the species, and how they are treated. Animals used as photo props may be captured from the wild (e.g. [17, 21–23]), physically restrained when they are not on-show, kept in poor conditions (e.g. [17]) and, for the safety of the human handlers, subject to having their claws or teeth removed or clipped (practices that can result in death of the animal [19]). Visitors are often largely unaware of such negative impacts [13], and the taking and sharing of such images in a public domain (such as on social media) may promote desire among those that view the images to attend such attractions themselves [24]. Zoos and animal rescue centres also often offer opportunities for close encounters with captive-bred individuals of wild animals (as ‘ambassadors’), presented by a trained handler in a setting that is usually intended to raise awareness and to build support for species protection. But without context these images cannot always be distinguished from those where animals are poorly treated and/or may have been taken illegally from the wild. There are concerns that these images may also propagate the photo-prop industry and encourage desire for inappropriate and/or illegal exotic companion animals [25–27]. Consequently, a number of organisations and groups advise against taking, and particularly, sharing such images on social media regardless of their provenance (e.g. [28, 29]).

**Fig 1:**
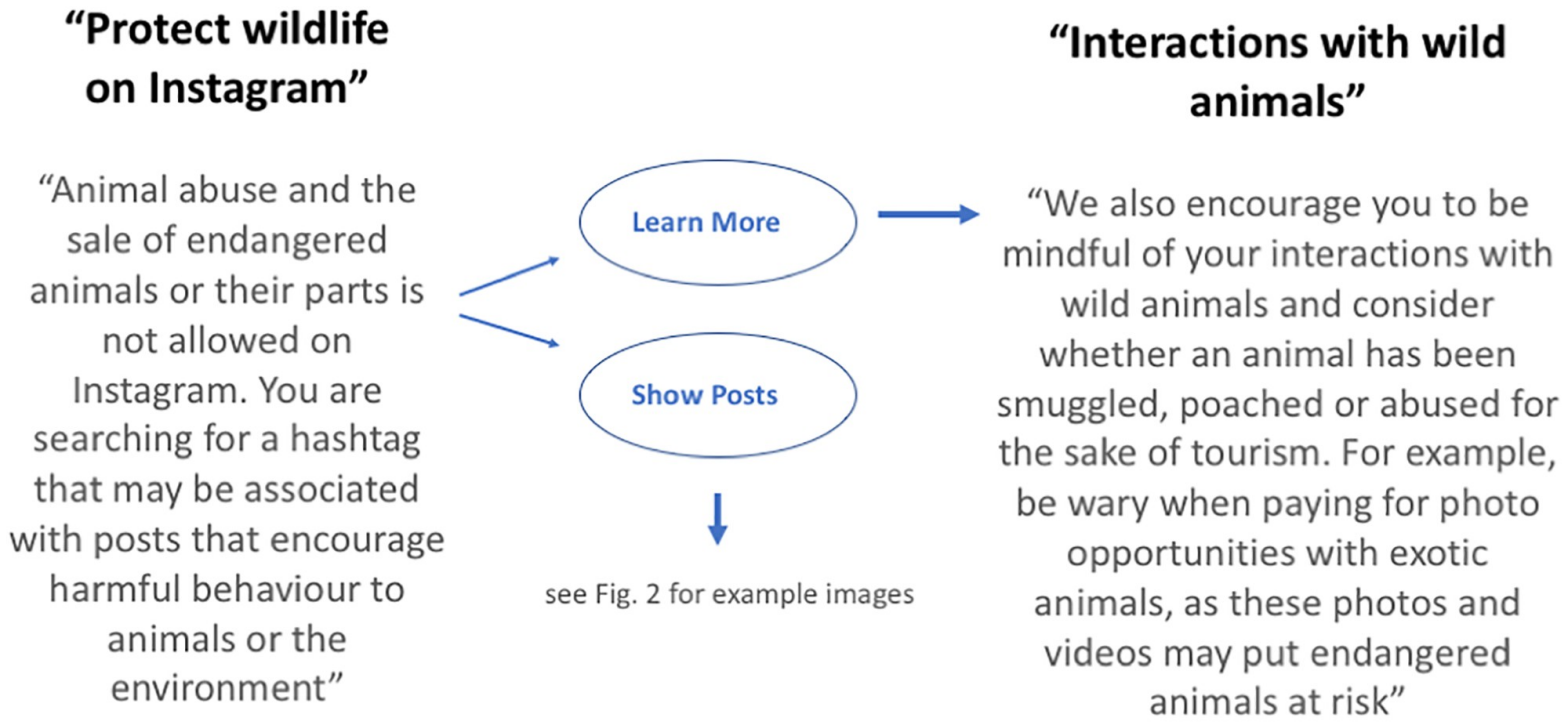
Instagram’s wildlife selfie pop-up alert system showing the warning presented when users search for wild animal selfie hashtags (e.g. #elephantselfie) (left), the follow-on options given (middle), and an extract from the information provided (right) when users select ‘Learn More’.

In an attempt to protect captive wild animals from mistreatment, and to raise awareness of the potential conservation and welfare issues associated with tourist attractions offering photo opportunities with wild animals, Instagram introduced a pop-up alert system (Fig 1) that is triggered when users search for wild animal selfie hashtags using Instagram’s search tool. Hashtags are words or phrases preceded by the symbol # that are used to tag content uploaded to social media. For example, ‘#slothselfie’ might be used by someone posting an image of a selfie photograph with a sloth, which then enables any other user searching for selfies with sloths to find that particular post. The aim of the initiative was not to block access to the posts but to educate users on the welfare issues that may underlie a seemingly benign animal encounter [30]. The warning provided also includes a link to further information about wildlife exploitation (Fig 1) to help users understand more about the problem. Instagram’s wildlife selfie alert initiative was implemented in December 2017 (more than 5 years ago to date). However, thus far (as far as we are aware) there has been no formal assessment of the policy’s effectiveness. In the long-term, the intended effect of the warning might conceivably (and usefully) have been to discourage the posting of wildlife selfies and thus to prevent or reduce imitation behaviours and the taking of wildlife selfies in the first place. These type of long-term behaviour changes are hugely challenging to monitor and the relevant data are rarely available (c.f. [31]). However, a more immediate (and measurable) effect might be expected on the perceptions and attitudes of the viewers that are presented with the warning prior to viewing a wildlife selfie post. We hypothesized for example that an informed viewer having read the warning might respond more negatively to the post (i.e. be less likely to ‘like’ a post, or more likely to express negative sentiments in the comments). Although there is also a risk that such messaging has the unintended consequence of stimulating greater interest in wildlife selfie posts (the so-called Streisand effect [32]) we considered this unlikely for these type of posts (in contrast with, for example, a desire to watch violent content, c.f. [33]).

We used Instagram posts depicting people in direct contact with elephants, a charismatic and popular species, and one of the most numerous captive-kept wild animals in WTAs [13], as a case study. The training required to ensure that tourists interacting with elephants are safe is described as being particularly cruel [34] and the conditions that elephants are kept in have been found, in many cases, to be inadequate in terms of animal welfare. Elephants used in tourist attractions are sometimes kept on chains, on concrete ground, and in the absence of social contact with other elephants [34, 35]. Riding elephants may cause elephants injury [36] but is still offered in some countries. Elsewhere, facilities offer tourists the opportunity to wash elephants in the river, to touch and to ‘hug’ their trunks, and to ‘cuddle’ juvenile elephants. These activities tend to be perceived as an ethical alternative to riding but can cause stress to elephants (e.g. [37, 38]) and are still dependent on the same type of training of young animals necessary to ensure (for the safety of visitors) that they can be controlled by their handler [35, 39].

A preliminary scoping study revealed that Instagram’s wildlife selfie alert was triggered inconsistently among posts and so we were able to compare viewer response to posts using a hashtag that triggered the warning with those that used hashtags that did not trigger the warning. We posed the following research questions:

1. What proportion of hashtags related to elephant selfies trigger Instagram’s warning?
2. Comparing elephant selfie posts that used hashtags that did trigger the warning with those that did not, is there any difference in:
  - post content
  - popularity of posts
  - perceptions or attitudes of viewers (as revealed by their comments)?

Overall, our aim was to assess how widely, and how effectively, the Instagram alert system is implemented and how viewers’ response to posts might be influenced by it. Ultimately, we sought to inform refinements of this initiative as well as the design and implementation of other such social media initiatives intended to benefit the welfare of wild animals, and the status of their populations, through encouraging appropriate relationships between people and the wild animals that they interact with.

## Methods

### Definition of an elephant selfie

**Fig 2:**
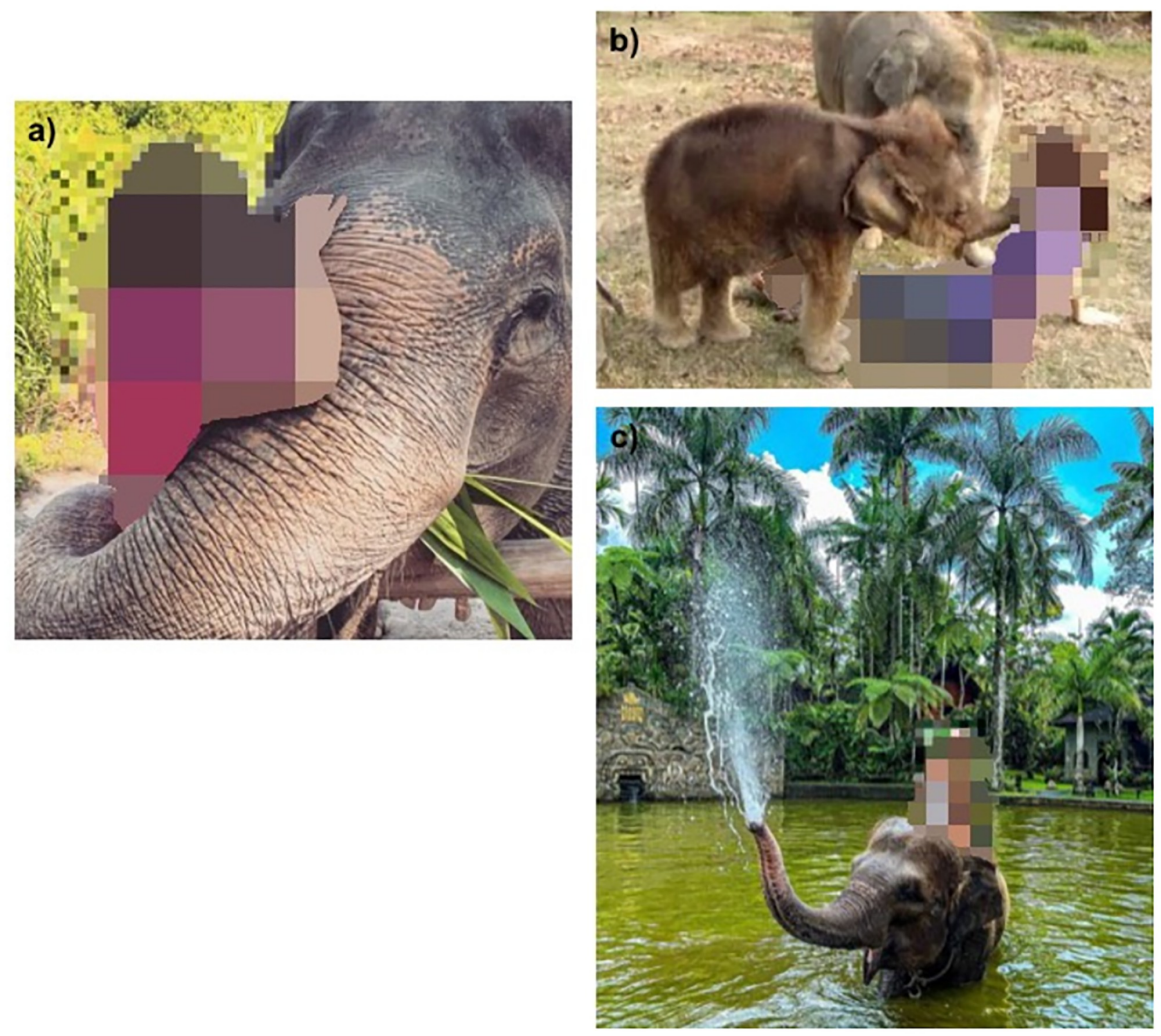
Example Instagram screenshots showing ‘elephant selfie’ images using the hashtags #elephantselfie (a, b), and #elephantride (c).

For the purpose of this study, we defined an ‘elephant selfie’ as an image of a person photographed in direct contact with an elephant, or standing in close proximity to an elephant (Fig 2), where ‘direct contact’ meant either touching, feeding, washing, or riding an elephant, and ‘close proximity’ was considered to be approximately within ‘touching distance’. We included photographs taken as a ‘selfie’ by the person shown in the image, and photographs taken by someone else (the latter may also be refered to as a ‘two shot image’[26]). This definition of wildlife selfies differs from the common meaning of the term ‘selfie’ (where the photo is taken by the person in the photo themselves) but follows standard usage and understanding of the term ‘wildlife selfie’ (as in, for example, [29]).

Our focus was specifically on visitors to wildlife tourist attractions, so we excluded images in circus settings, and those where the person in the image was clearly an elephant keeper or carer (indicated by the uniform worn, or the text accompanying the post). We also excluded images showing wild elephants in the background of the image (i.e. where the person was in an open safari vehicle), or images that had been digitally manipulated to make it appear that a person was close to an elephant, because these did not depict people in direct contact with elephants.

### Triggering of Instagram’s wildlife selfie alert

To determine how often, and how effectively, Instagram’s wildlife selfie alert is triggered, we initially searched Instagram, using the platform’s search tool on the Explore page, for as many hashtags as we could find associated with elephant selfies using words related to our definition (e.g. #elephantselfie, #elephantselfies, #eleselfie, #elephantwashing, #elephantrides, and others) and recorded whether a subsequent search for posts under each identified hashtag triggered the warning. Additional relevant hashtags were obtained by snowball sampling [40], using all hashtags suggested by Instagram under each term entered in the search bar. We included hashtags in the final count only if they led to one or more ‘elephant selfie’ posts (as defined above), and excluded those that were associated exclusively with posts by organisations (e.g. animal welfare groups) advocating against close interactions with elephants (e.g. #elephantridingiscruel), where images were included only for informative purposes. To account for frequency and recency of use, we recorded post volume (the number of posts that each hashtag had been used in) obtained from the search results provided by Instagram, and if the term had been used in posts posted since 2017 (i.e. since the introduction of Instagram’s wildlife selfie alert). Post volumes were sometimes reported by Instagram only as approximate values so statistical comparison was not possible. All searches were completed between 28.09.21 and 20.10.21.

### Comparison of elephant selfie posts on Instagram using hashtags that do and do not trigger the warning

To explore in more detail the implementation, and effect of, Instagram’s wildlife selfie alert we used a comparative study design in which we compared posts, and viewer response to posts, between pairs of similarly worded hashtags, one of which triggered the warning, and one of which did not. Our sample of hashtags was not random but was selected specifically to include terms that triggered the warning, that had a comparable (similarly worded) hashtag that did not trigger the warning, and that were used in a large number of Instagram posts. This design meant that inferences were limited to the specific hashtags used in the study but allowed any differences detected to be attributed to the (potential) triggering of the warning (rather than to differences in the terms used) and ensured a sufficient number of posts (and comments to posts) for estimation of post metrics and sentiment scores (below). Three pairs of similar hashtags were suitable for comparison: #elephantselfie and #elephantselfies, #elephantride and #elephantrides, #elephanthugs and #elephantcuddles; in each case, the first of each pair triggered the warning and the second did not (see Table 1).

**Table 1:** Categories used to describe posts. ‘Situation’ refers to whether the elephant is captive (C) or wild (W); the presence of people in the image is denoted by Y (yes) or N (no), and the role of the people in the image as either ‘in close proximity to, or touching the elephant’ (close/touching) or ‘observing from a distance’ (observing).

Category	Description	Situation	Presence/role of people
Elephant selfie	an image of a person photographed in direct contact with an elephant, or standing in close proximity to an elephant (see text)	C	Y (close, touching)
Captive elephant	captive-held elephants photographed alone, i.e. painted elephants, or elephants clearly in a zoo or with a keeper or carer	C	Y or N
Elephant safaris	wild elephants shown with people in the foreground of the image, i.e. in an open safari vehicle	W	Y (observing)
Wild elephants	wild elephants pictured in natural habitats, with no people in the image	W	N
Advocacy	elephant selfie images included in a post advocating against direct interactions with elephants	C	Y (close, touching)
Irrelevant	all other images, e.g. toy elephants, drawings of elephants, or any other image not showing a live elephant	NA	NA

#### Post content

To assess whether Instagram’s wildlife selfie alert, on average, captures the content that it was intended to, we compared post content in relation to the presence/absence of hashtags that triggered the warning. For each hashtag, two independent observers viewed and categorised posts shown on a mobile device (in the order presented on the device, under ‘top’ where the option was available). Each post shown was counted as being within one of six pre-defined categories, as defined in Table 1. These categories were intended to broadly describe the situation of the elephant in the image and the presence/role of the people in the image, and to be representative of the type of animal-tourist images that might be seen on social media generally. Viewing was terminated when most posts were dated 2017 or earlier, were categorised as ‘irrelevant’, or (in the case of #elephantride/s) when a minimum of 400 posts had been viewed.

Because posts are not necessarily shown in the same order on different devices or under different user accounts, we used chi-squared tests to test for broad agreement between observers in the relative distribution of content categories under each hashtag rather than tests of inter-observer agreement based on paired observations. This approach means that any difference detected may be due *either* to differences between observers (lack of observer reliability or agreement) *or* inconsistency between devices or Instagram accounts. In all cases, we detected no difference between observers (chi-squared test, p > 0.08 in all cases; six tests performed separately, Bonferroni-corrected critical p value = 0.008).

#### Viewer response

To explore the effect of the use of hashtags that triggered the warning on viewer response to elephant selfie posts specifically, we, first, quantified the popularity of posts, and, second, analysed viewer comments on the posts using sentiment analysis. For this analysis, we used only those posts that conformed to our definition of an elephant selfie (above) and that had been posted in 2018 or later. For comparative purposes, posts that used more than one of the six hashtags were excluded. For each of the six hashtags, we manually collated data for each post including the date that it had been posted, the country where it was posted (obtained from the geo-location provided, or inferred from the text contained within the post), and the number of likes that the post received. Still image posts and video posts were treated in the same way and we did not distinguish between them. We used the number of likes as an indicator of post popularity to reflect a combination of the number of times a post was viewed *and* liked; this is not the same as engagement rate (calculated as the number of likes as a percentage of the number of followers) which measures popularity *within* the audience that ordinarily follow the posters account. Both metrics have weaknesses. The disadvantage of the number of likes as a measure of popularity is that it provides no information on the number of viewers that did not like the post. Whereas engagement rate does not account for the number of views received via searches for hashtags and so may significantly overestimate engagement for posts using popular hashtags. On balance, and mindful of data accessibility and privacy issues (and the fact that the number of followers can only be obtained by accessing personal accounts), we considered number of likes most suitable for the purposes of this study. Posts were retrieved in the order in which they were presented on a desktop computer, and the search for relevant posts continued until either there were no more posts available, or most were irrelevant or dated 2017 or earlier (up to a maximum of approximately 100 posts). For the hashtags that triggered a warning, only a limited number of posts were shown on a desktop computer, in which case data for the remaining posts were manually copied from a mobile device. Our sample of posts, thus represents a subset of all possible posts but includes the ‘top’ (the most highly viewed) and the most recent posts shown.

Finally, viewer comments were manually copied from each post, and comments on all posts combined in a single document for each of the six hashtags. Documents were saved as plain text files for analysis, where lines of text corresponded to individual comments. For this analysis we were only able to retrieve comments from posts shown on a desktop computer (see above). We quantified the sentiment of comments to posts using each of the six hashtags, using a sentence-level lexicon-based approach. A sentence-level approach assigns a sentiment score to each sentence (comment) based on the sum of the scores of all meaningful (scorable) words within the comment (i.e. all words that exist in the lexicon and have an associated score, taking account of the word count used). We used the “sentimentr” package [41] in R to assign sentiment scores, which is able to account for valence shifters and modifiers in the text and allows the incorporation of emojis. Emojis enhance, and modify the meaning of, the text [42] and so their inclusion not only adds information from the comments based only on emojis, but also improves the accuracy of sentiment scoring compared to using the linguistic text alone [43, 44]. Prior to analysis names, dates, profile pictures, identities, personal comments irrelevant to the context of the image (e.g. comments referring to personal circumstances or appearance), advertising (e.g. of other local sanctuaries), and the word ‘instagram’, were manually identified and removed. Foreign text was replaced with English language using google translate where possible. Emojis were separated into individual symbols so that each counted as a single ‘word’, and replaced with identifiers that can be recognised by sentimentr, using the “textclean” [45] package in R. The sentimentr package provides a sentiment score between -1 and +1 for each scorable word, and includes an emoji lexicon (with comparable scores for each listed emoji, based on [42])–we combined these two into a new bespoke lexicon and obtained sentiment scores for each comment (based on the text and emojis in each comment) using the ‘sentiment_by’ function in sentimentr. Mean sentiment scores were calculated for the complete comment text for each of the six hashtags by averaging the individual sentence scores, and standard errors of the means estimated using the package “plotrix” [46]; in addition, the number of negative, neutral, and positive sentence sentiment scores were counted for each hashtag. In addition, to provide context for sentiment scores, we identified the most frequently occurring words in the comment text (including emojis as words) for each of the six hashtags, using the text mining package “tm” [47, 48]. Stopwords were excluded, except for the word ‘not’ due to its potential importance as a valence shifter; where not was identified as a frequently occurring word we examined the original comment text to determine the context that it was used in.

### Ethical considerations

Our study involved covert observation [49]; however, we used only data (images shown, post metrics, and comments) that were publicly available on the Instagram platform, and posted on public accounts. No automated data collection methods were used, and data were collected and analysed within the terms and conditions of Instagram (www.instagram.com). We did not engage in deceptive practice, and did not engage directly or otherwise with Instagram users (either those posting images or commenting on images). This was an independent study that was not formally linked with an academic institution; therefore, none of the authors had access to an institutional ethics review body. In the absence of formal external ethical review we relied on published guidelines and principles for the use of social media data in research. It is noteworthy that this is a rapidly evolving field, there is currently a lack of *standardised* guidance, review boards often do not have appropriate expertise, and there is disagreement amongst authors publishing on this topic (see e.g. [50], and references therein). Bearing this in mind, we took two standpoints. Firstly, to protect the identity of individual Instagram users (and thus to ensure annonymity), user names were not recorded, and user names, profile pictures, and any names and/or personal details were removed from text files created and saved for analysis (this is in accordance with ethical research practices as outlined in [51] and [52]). Secondly, because commenters were not identified individually, and data deriving directly from comments (with the exception of four annonymised quotes used for illustrative purposes, section 3.4) are presented here only in aggregate form (single words and sentiment scores), we considered that informed consent was not necessary (this is in accordance with [52]).

### Statistical analysis

All statistical analyses were carried out in R (version 4.1.2; [53]). For left-skewed data, we report medians, and use a robust coefficient of variation (RCV_Q_, based on the median and the interquartile range [IQR/median * 0.75] [54]) as a measure of relative dispersion within samples. We used Pearson’s chi-square test to test for a difference in post content between posts that used hashtags that triggered Instagram’s alert system and those that used hashtags that did not trigger the alert, for an association between the origin (country) of a post and the hashtag used, and for differences in the proportion of positive, negative, neutral comments between pairs of hashtags. We compared popularity (quantified as the number of likes) between each pair of hashtags using non-parametric two sample Mann-Whitney U Tests, and used Welch’s two sample t tests to test for a difference in mean sentiment scores between pairs of terms. In all cases, pair-wise comparisons were carried out separately and Bonferroni corrections used to account for multiple tests. Effect sizes were quantified, for non-parametric tests, using Hodges-Lehmann (HL) estimator (which is the sample median of all cross-sample pairwise differences) and, for parametric tests, using the mean difference; we used non-standardised effect sizes because these are straightforward to interpret for the parameters (number of likes and sentiment scores) used. Violin plots, used to depict the distribution and range of the data, were drawn using the “vioplot” package in R [55].

## Results

### How many hashtags related to elephant selfies trigger Instagram’s warning?

We identified a total of 244 hashtags associated with Instagram posts that included various types of elephant selfie (Table 2). Three-quarters of the hashtags identified (n = 183) had been used since 2017. When searched for, 2% of all hashtags identified (n = 5; 2.7% of those that had been used since 2017) triggered Instagram’s warning. Post volumes for hashtags that triggered the warning were > 1,000 in all cases (maximum = 156,169, Table 2). Post volumes for hashtags that did not trigger the warning were hugely variable (range 1 –> 10,000, n = 239, Table 2, S1 Appendix) such that whilst 159 (66.5%) hashtags that did not trigger the warning had post volumes < 10, 18 (7.5%) had post volumes > 1,000, and two (0.8%) had post volumes > 10,000.

**Table 2:** Hashtags associated with elephant selfie posts on Instagram. Data shown are the post volume (the number of posts in which the hashtag was used), whether Instagram’s pop-up alert was triggered (Y = yes, N = no), and whether any of the posts had been posted (i.e. the hashtag had been used) since the warning was introduced in 2017. Not all posts conformed to our definition of an elephant selfie; hashtags were included if at least one post using it was relevant (see footnote). Only hashtags with a post volume > 500 are shown here, the full set of identified relevant hashtags is provided in S1 Appendix. Neither the list of hashtags here, or the full list of hashtags given in S1 Appendix, is intended to be exhaustive. Bold text indicates hashtags used for further analysis.

Search term	Post volume	Warning triggered	Used in posts since 2017
**#elephantselfie**	**22269**	**Y**	**Y**
**#elephantselfies**	**705**	**N**	**Y**
**#elephantride**	**82601**	**Y**	**Y**
**#elephantrides**	**16922**	**N**	**Y**
#elephantriding	29952	Y	Y
#elephantsanctuary	156169	Y	Y
#selfiewithelephant	654	N	Y
#elephantsanctuaryphuket	5884	N	Y
#elephantbath	12752	N	Y
#elephantsanctuarychiangmai	9436	N	Y
#elephantsanctuarythailand	1538	N	Y
#elephantride 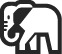	1000+	N	Y
#elephantriders	1000+	N	Y
#elephantrider	1000+	N	Y
#elephantriding 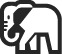	1000+	N	Y
#elephantsanctuarybles	1000+	N	Y
#elephantbathing	5000+	N	Y
#elephantbathtime	500+	N	Y
#elephantwashing	1000+	N	Y
#bathingwithelephants	1000+	N	Y
#eleselfie	500+	N	Y
**#elephanthugs**	**1000+**	**Y**	**Y**
**#elephantcuddles**	**1000+**	**N**	**Y**
#feedingelephants	5000+	N	Y
#elephantfeeding	5000+	N	Y
#bathingelephants	1000+	N	Y
#washingelephants	1000+	N	Y

The following hashtags were used exclusively in posts advocating against elephant rides or direct contact with elephants: #elephantridesarecruel, #elephantridingistorture, #elephantridingsucks, #elephantridingisnotcool, #elephantridingisabuse, #elephantridingiswrong, #elephantridingiscruel, #elephantrides❌.

### Comparison of elephant selfie posts on Instagram using hashtags that do and do not trigger the warning

We initially viewed a total of 2,512 Instagram posts in order to describe post content in relation to the presence/absence of hashtags that trigger the warning (n = 468 using the term #elephantselfie^!^, 225 #elephantselfies, 871 #elephantride^!^, 487 #elephantrides, 193 #elephanthugs^!^, and 268 #elephantcuddles; where the terms marked with an exclamation mark triggered Instagram’s warning and those without did not). Analyses of post popularity were carried out on a subset of viewed posts that conformed to our definition of elephant selfies (above). Thus our main dataset comprised a sample of 440 elephant selfie posts, posted between 2018 and 2021, that used at least one of the six hashtags (n = 57 used the term #elephantselfie, 48 #elephantselfies, 122 #elephantride, 16 #elephantrides, 97 #elephanthugs, and 96 #elephantcuddles, excluding four posts that used more than one of the hashtags). Location could be inferred for 403 (91.6%) of elephant selfie posts (S2 Appendix). Elephant selfie posts originated from 15 different Asian and African countries, and the USA, but were predominantly from Thailand (50% posts, n = 218), India (14.2%, n = 62), and Indonesia (11.9%, n = 52). Posts from India were most likely to be associated with #elephantride or #elephantrides (82.3% of India origin posts, n = 51), whereas those from Thailand (51.8% of Thai origin posts, n = 113) and Indonesia (59.6% Indonesian origin posts, n = 31) were most likely to be associated with #elephanthugs or #elephantcuddles (association between country and hashtag: χ^2^ = 93.70, df = 6, p<0.001, countries combined as Thailand, India, Indonesia, ‘Other’, and hashtags combined as #elephantride/s, #elephantselfie/s, #elephanthugs/cuddles). Because viewer comments were only retrieved from posts shown on a desktop computer (see Methods), sentiment scores were calculated for a reduced dataset comprising a total of 221 posts (n = 12 using the term #elephantselfie, 48 #elephantselfies, 27 #elephantride, 16 #elephantrides, 21 #elephanthugs, and 96 #elephantcuddles).

### How does post content differ among posts using hashtags that do and do not trigger the warning?

**Fig 3:**
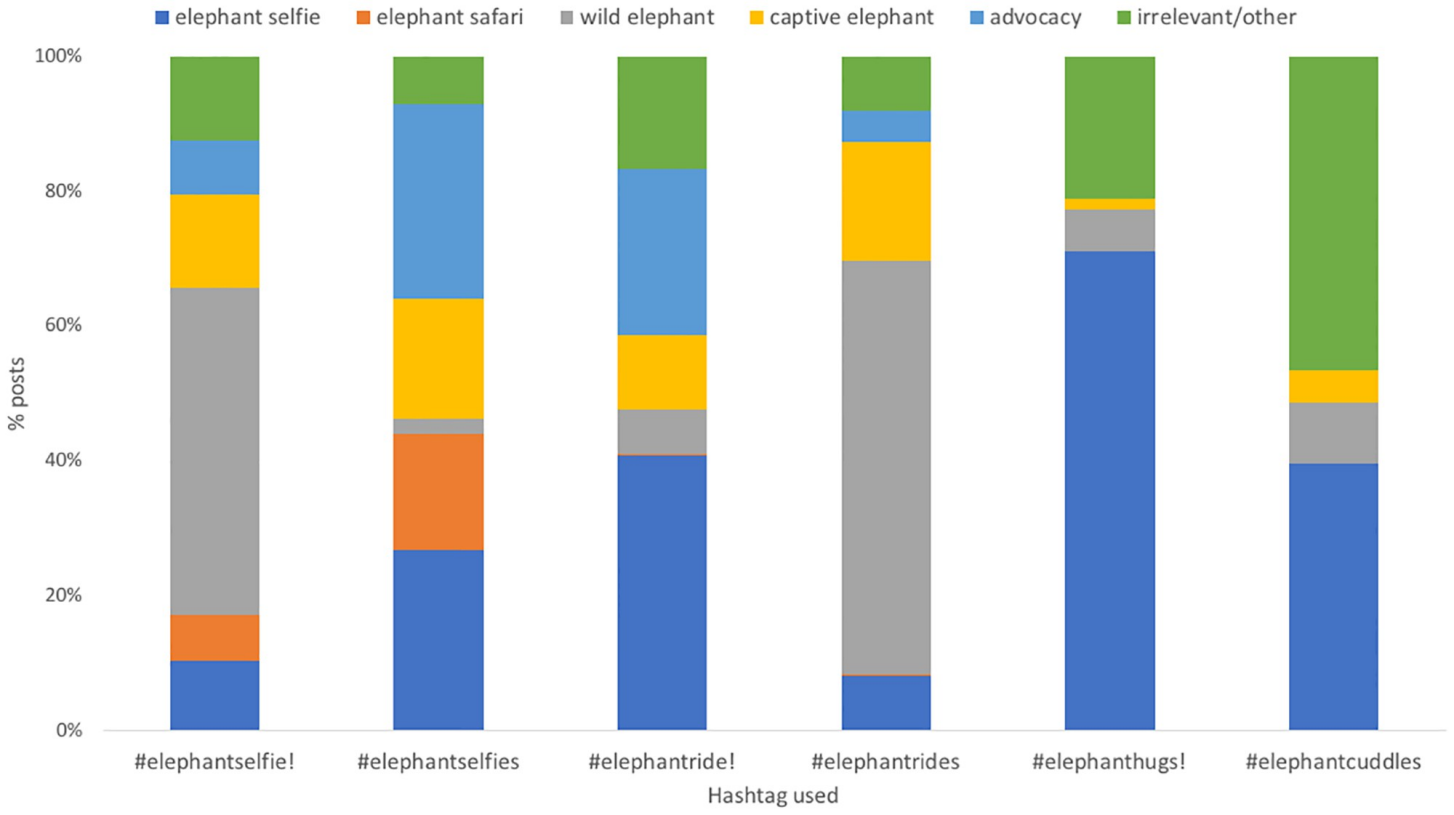
Types of post using each of six hashtags, where the first of each ‘pair’ (#elephantselfie-#elephantselfies, #elephantride-#elephantrides, #elephanthugs-#elephantcuddles) triggers Instagram’s warning (marked by an!) and the second does not. Relative proportions of post types were statistically significantly different between pairs of terms in all cases; n = 468, 225, 871, 487, 193, 268, respectively. Content categories defined in Methods and Table 1.

For the pair of terms #elephantride and #elephantrides, posts using #elephantride (which did trigger the warning) comprised a larger proportion of ‘elephant selfie’ posts (40.6%, n = 354 of 871 posts) than did posts using #elephantrides (8.0%, n = 39 of 487) (which did not trigger the warning); the latter comprised predominantly (61.4%, n = 299 of 487) ‘wild elephant’ posts (Fig 3). A similar trend in elephant selfie posts was apparent between the terms #elephanthugs (which did trigger the warning) and #elephantcuddles (which did not): posts using #elephanthugs comprised 71.0% ‘elephant selfie’ posts (n = 137 of 193 posts), whilst posts using #elephantcuddles comprised 39.6% ‘elephant selfie’ posts (n = 106 of 268). For #elephantcuddles, the majority of posts using the term were categorised as ‘irrelevant’ (46.6%, n = 125 of 268 posts; we did not attempt to describe this category in any further detail but noted that several posts showed images of soft toy elephants, some photographed alongside human babies). The pair of terms #elephantselfie and #elephantselfies, however, showed the opposite trend with posts using #elephantselfie (which did trigger the warning) comprising a smaller proportion of ‘elephant selfie’ posts (10.3%, n = 48 of 468 posts) than did posts using #elephantselfies (26.7%, n = 60 of 225) (which did not trigger the warning); in this case, the posts using the term that triggered the warning (#elephantselfie) comprised predominantly (48.5%) ‘wild elephant’ posts (n = 227 of 468). Advocacy posts comprised a relatively large proportion (28.9 and 24.8%, respectively) of posts using the hashtags #elephantselfies and #elephantride (terms that did not, and did, trigger the warning, respectively).

Overall, all six hashtags were used in all types of post, with the exception of #elephanthugs and #elephantcuddles, which were not used in either ‘elephant safari’, or ‘advocacy’ posts. However, although there were statistically significant differences in the relative proportions of types of post between pairs of hashtags (chi-squared tests, p < 0.001 in all cases, Bonferroni-corrected critical p value for 3 tests = 0.017), there was no consistent pattern between those that did trigger Instagram’s warning and those that did not (Fig 3).

### Is there any difference in the popularity of ‘elephant selfie’ posts using hashtags that do and do not trigger the warning?

**Fig 4:**
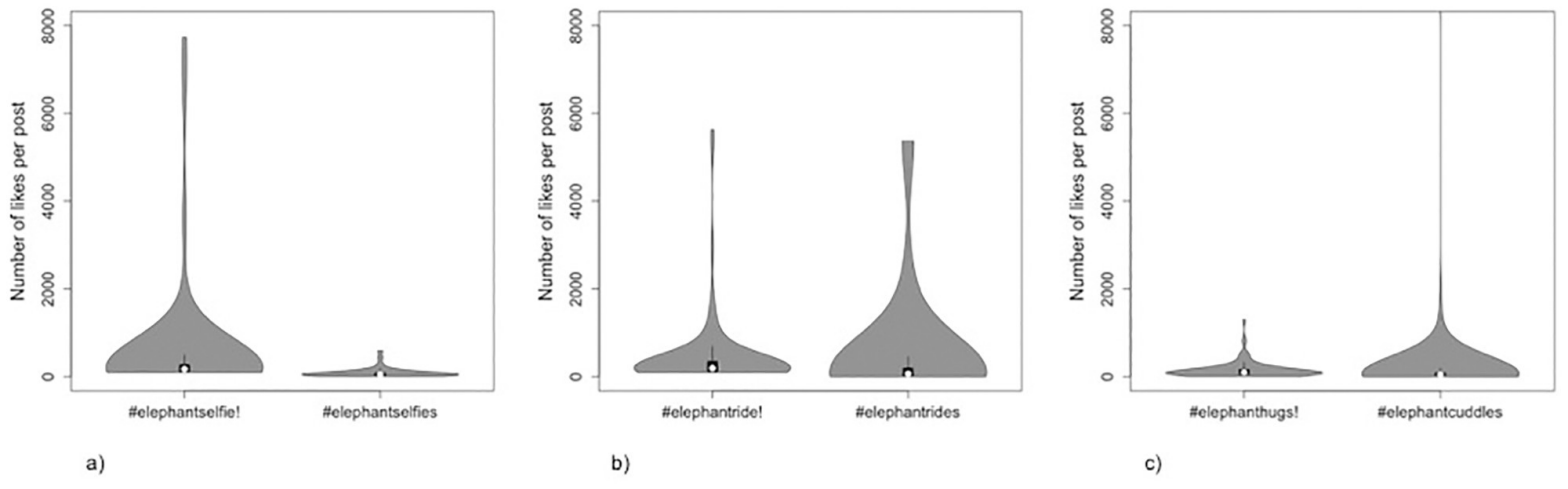
Violin plots showing popularity (as measured by number of likes) of elephant selfie posts using each of six hashtags, where the first of each pair of terms—a). #elephantselfie and #elephantselfies, b). #elephantride and #elephantrides, c). #elephanthugs and #elephantcuddles—triggers Instagram’s warning (marked by an!) and the second does not. Circles depict the median (RCV_Q_ = 0.64, 0.88, 0.86, 1.94, 0.91, 0.97, respectively); maximum value for #elephantselfie not shown to improve visual comparison.

Popularity of elephant selfie posts (measured as the number of likes) was highly variable within and between hashtags, and heavily-skewed towards lower values (i.e. most posts received relatively few likes, Fig 4). Median number of likes across hashtags ranged between 51.5 and 194.5, whilst maximum values for five of the six hashtags exceeded 1,000 (> 24,000 for posts using #elephantselfie, Fig 4); for all hashtags, within-term robust coefficients of variation were relatively high (RCV_Q_ = 0.64–1.94, summary data in S3 Appendix). The number of likes for posts using hashtags that triggered Instagram’s warning was statistically significantly different from the number of likes for those that did not, for all pairs of terms (p<0.01, in all cases; Bonferroni-corrected critical p value for 3 tests = 0.017), but, contrary to expectation, median likes for posts using hashtags that triggered the warning exceeded median likes for those that did not (i.e. were more popular), in all cases. Median cross-sample pairwise differences (HL estimators) were 112, 105, and 38 for #elephantselfie/s, #elephantride/s, and #elephanthugs/cuddles, respectively (i.e., on average, a post using the hashtag ‘elephantselfie’ received 112 more likes than did a post using the hashtag ‘elephantselfies’).

### Is there any difference in the perceptions or attitudes of viewers (as revealed by their comments) to posts using hashtags that do and do not trigger the warning?

**Fig 5:**
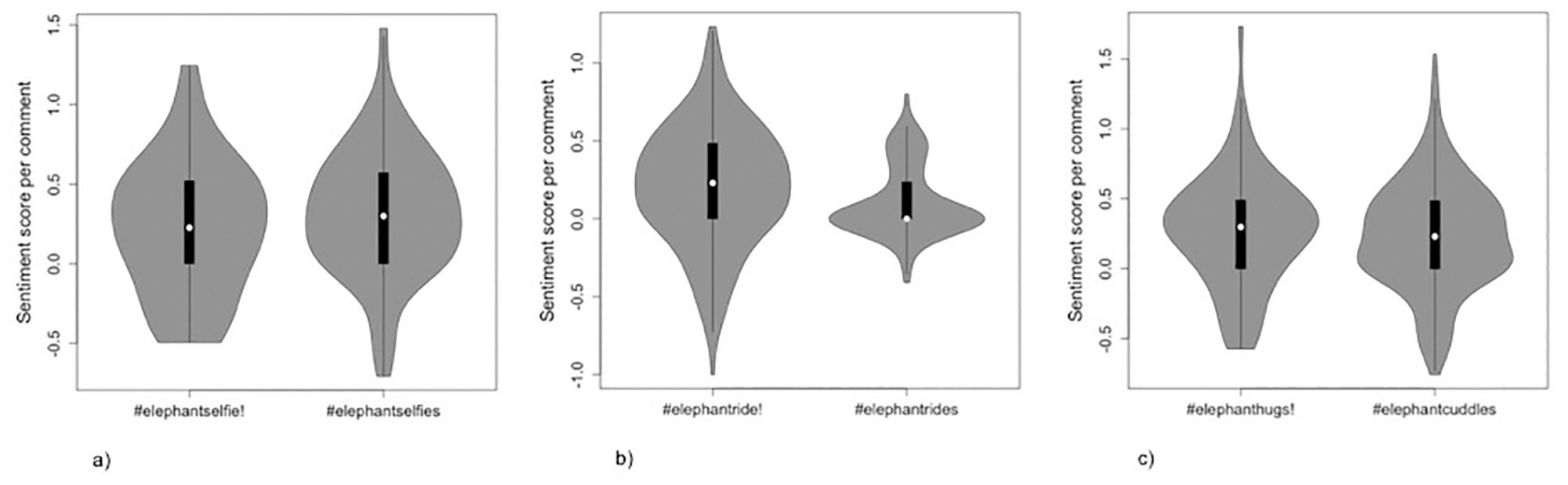
Violin plots showing the distribution and range of sentiment scores for comments on elephant selfie posts using each of six hashtags, where the first of each ‘pair’ of terms—a). #elephantselfie and #elephantselfies, b). #elephantride and #elephantrides, c). #elephanthugs and #elephantcuddles—triggers Instagram’s warning (marked by an!) and the second does not. Circles depict the median, the bars depict the inter-quartile range.

For posts using all hashtags, sentiment scores of comments were approximately normally distributed and ranged between a minimum of -1.0 – -0.41 and a maximum of 0.8–1.73 (i.e. all contained both negative and positive comments). Mean sentiment scores were positive in all cases, with values between 0.11 and 0.32. Mean sentiment scores did not differ significantly between comments to posts using #elephantselfie (that triggered the warning) and those using #elephantselfies (that did not trigger the warning; p = 0.165), or between those using #elephanthugs and #elephantcuddles (that did and did not trigger the warning, respectively; p = 0.397, Fig 5, Table 3); however, mean sentiment for comments to posts using #elephantride was statistically significantly higher (more positive) than for comments to posts using #elephantrides (i.e. comments to posts that used a hashtag that triggered the warning were more positive than those that did not; t = 2.83, df = 237, p = 0.005, Bonferroni-corrected critical p value for 3 tests = 0.017, mean difference = 0.10, Fig 5, Table 3). There was also a statistically significant association between the polarity of the sentiment score and whether or not the hashtag used triggered the warning for comments to posts using #elephantride/s (p <0.001; but not for those using #elephantselfie/s or #elephanthugs/cuddles, p = 0.119, 0.093, respectively, Bonferroni-corrected critical p value for 3 tests = 0.017)–although in this case, comments to posts using the hashtags that did trigger the warning (#elephantride) appeared to have more negative *and* positive scores (i.e. more polarised scores) rather than more positive scores specifically (there was no association between the presence of the warning and the proportion of negative and positive comments [p = 1.0], but there was a statistically significant association between the presence of a warning and the proportion of polarised and neutral comments [p < 0.001]).

**Table 3:** Sentiment scores for comments to posts using each of six hashtags, where the first of each ‘pair’ of terms triggers Instagram’s warning and the second does not. Sentiment scores (assigned, based on text and emojis combined, using the “sentimentr” package (Rinker 2021) in R), reported as mean, SE, and median, and percentage negative, neutral, and positive; sample size (n) is given as the number of posts, the total number of comments for all posts using each of the hashtags combined, and the number of words used in the analysis.

#	Warning	n posts	n	Sentiment score	Sentiment score
			comments, words	mean (SE), median	(%) neg, neut, pos
#elephantselfie	Y	12	49, 217	0.220 (0.060), 0.226	24.5, 6.1, 69.4
#elephantselfies	N	48	108, 718	0.319 (0.038), 0.299	13.0, 13.0, 74.0
#elephantride	Y	27	248, 2487	0.206 (0.024)*, **0.229**	**23.4, 6.9, 69.7** **
#elephantrides	N	16	81, 660	0.107 (0.025)*, **0.000**	**11.1, 56.8, 32.1** **
#elephanthugs	Y	21	83, 483	0.271 (0.044), 0.299	21.7, 4.8, 73.5
#elephantcuddles	N	96	384, 2700	0.230 (0.020), 0.231	18.7, 13.3, 68.0

* statistically significantly different (p < 0.01);

** statistically significantly different (p < 0.001).

The most frequently occurring word (or symbol) in the comments to posts for all hashtags was a ‘smiley face with heart eyes’, and the second or third most frequent a heart symbol (Table 4). Overall, for all hashtags, words and symbols tended to be positive in nature (including, for example, the words ‘love’, ‘amazing’, ‘beautiful’), with some exceptions for comments to posts using the two hashtags associated with riding elephants (#elephantride and #elephantrides). However, symbols depicting more negative emotions (such as anger [‘angry red face’] and sadness [‘crying face’]) occurred in comments to both elephant riding hashtags regardless of whether or not they triggered Instagram’s warning (Table 4). The same was true of the word ‘not’ which also occurred in comments to posts using both elephant riding hashtags, and was used, in more than half of all occurrences (n = 11 of 20 occurrences, and 7 of 10 occurrences in comments to #elephantride and #elephantrides, respectively) in phrases like “…tourists benefit, *not* the elephants”, “should *not* be encouraged”, “*not* your entertainers”, “*not* a pet“, “*not* here to serve us“.

**Table 4:** Most frequently occurring words in comments on elephant selfie posts using each of six hashtags, where the first of each ‘pair’ of terms—a). #elephantselfie and #elephantselfies, b). #elephantride and #elephantrides, c). #elephanthugs and #elephantcuddles—triggers Instagram’s warning (marked by an!) and the second does not. Data are the frequency of occurrence of words and/or emojis appearing the comments, including only those with a frequency of occurrence of 10 or more. Stopwords were not included, except for the word ‘not’ which may be important as a valence shifter. Words and emojis with negative sentiment scores are shaded in the table.

#elephantselfie^!^	#elephantselfies	#elephantride^!^	#elephantrides	#elephanthugs^!^	#elephantcuddles
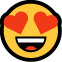 29	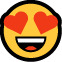 39	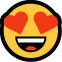 97	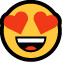 60	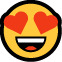 45	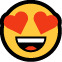 114
	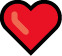 16	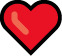 49	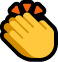 28	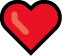 17	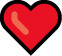 56
	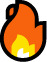 16	beautiful 26	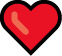 21		love 29
		not 20	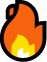 13		omg 17
		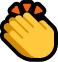 15	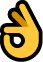 11		amazing 17
		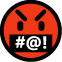 14	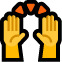 11		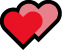 16
		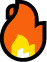 12	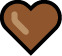 10		cute 15
		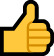 12	not 10		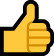 12
		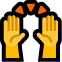 12	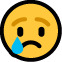 10		beautiful 11
		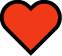 12			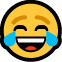 10
		wow 11			
		amazing 11			
		love 10			
		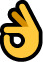 10			

## Discussion

Instagram and other social media platforms (such as YouTube) are currently entirely self-regulated [56]. As such, user-generated content posted on most social media platforms is usually subject to some form of acceptable standards or community guidelines derived by each individual platform (for Instagram, see https://help.instagram.com). Compliance, or indeed detection of non-compliance, is, in most cases, at least partly dependent on viewers identifying and flagging inappropriate content (e.g. [57]). In the case of wildlife selfies this is difficult because there is nothing in the image itself to suggest cruelty to nonhuman animals or negative conservation impacts on threatened species; neither is it the case that every image of a smiling human depicted alongside a wild animal is associated with detrimental impacts on animals at either an individual, population, or species level. To understand these issues requires much greater detail on where individual wild animals depicted are sourced from, the conditions that they are kept in (e.g. [17]), and the training that they are subject to, than is possible from a single image, as well as a much deeper understanding of their welfare requirements in captivity (e.g. [58]), the welfare impacts associated with handling [18], and the conservation status of the species in the wild (e.g. [21]). In this respect, Instagram should be commended for launching an initiative that attempts to educate users on some of the more subtle (or otherwise hidden) issues associated with the use of photo props in tourist destinations [30, 59] (see also [13, 60]) and of wild animal exploitation more broadly. However, to be genuinely informative, educational messages need to be visible to the correct target audience, easy to understand, sufficiently repetitive that they are noticed and remembered, consistent (to avoid ambiguity or confusion), relevant, and, in addition, users need to be receptive to the message [61–64].

Three key findings of this study suggest that Instragram’s wildlife selfie alert system as currently implemented is limited, inconsistent, and, in many cases, appears to be mismatched with the type of content that it was intended to highlight. Amongst 244 hashtags related to elephant selfies that we were able to identify, 98% failed to trigger Instagram’s alert system. Differences in hashtag terms that did and did not trigger the warning were extremely minor; for example, #elephantselfie did trigger the warning, #elephantselfies did not, yet both were associated with posts showing the types of wildlife selfie identified as being of potential concern (i.e. those showing close or direct contact with wild animals, cf. [65]). Conversely, #elephantselfie (which did trigger the warning) was associated, in almost half of all posts examined, with images showing only apparently wild elephants where the warning was irrelevant. The other two pairs of hashtags examined (#elephantride and #elephantrides, and #elephanthugs and #elephantcuddles) similarly failed to show any apparent pattern in associated content that might explain why the warning was triggered for one and not the other. Whilst some of the hashtags identified that did not trigger the warning were infrequently used, and our study was limited to elephant selfies, a brief search for other wildlife species featured in wildlife selfies on Instagram reveals similar inconsistencies: #koalaselfie (with 5,000+ posts), #slothselfie (22,600 posts), and #monkeyselfie (14,100 posts) all trigger the warning but the plural versions of these terms—#koalaselfies (100+ posts), #slothselfies (500+ posts), #monkeyselfies (1,000+ posts)—and a variety of other related terms, do not. Further, whilst not part of our initial study, *post hoc* observations revealed that the warning is triggered only when hashtags are used to search for images and is not triggered when posting images or when following a user account, meaning that users can view and interact with (‘like’ or comment on) posts without ever encountering the warning. Ultimately the warning does not prevent content from being posted.

The words and emojis used most frequently in post comments (Table 3), together with mean comment sentiment scores (which were > 0 in all cases, Table 2), suggest that elephant selfie posts included in the study were perceived as loveable, exciting, visually appealing, overall positive, and enviable experiences regardless of the hashtag used by the poster. Although, for each of the hashtags, there was some level of negativity in post comments (Table 2, Fig 5), there was no evidence that this was associated with the presence of Instagram’s warning. The effect sizes observed for popularity of posts (where posts using hashtags that triggered the warning appeared to be more popular than those using hashtags that did not trigger the warning) were small relative to the range of values recorded and likely not meaningful. That a statistically significant difference between mean comment sentiment scores (albeit also with a small mean difference) was observed only for posts using hashtags associated with elephant riding (where posts with the hashtag that triggered the warning received more positive comments on average than those with the hashtag that did not trigger the warning) suggests that this was an artefact of sampling rather than an effect of the warning per se. It is noteworthy, for example, that we were only able to include 16 posts that used the term #elephantrides. Further, the occurrence of some clearly negative words and emojis amongst those most frequently used in post comments, appeared to be associated with the act of riding elephants rather than presence of the warning. Overall, post comments suggested an apparent lack of broader awareness of the potential negative welfare impacts on elephants involved in alternative activities (‘hugging’, ‘cuddling’, and washing young and/or adult elephants), despite travel guidelines advising against such activities [66].

There are complexities associated with the long-tradition of elephant keeping in South East Asia (historically, for logging) in terms of the fate of those elephants already in captivity, and the livelihoods of their keepers (e.g. [67]) that are beyond the scope of this study. Nevertheless, our observations of social media posts, and viewer response to them, highlight inconsistencies between what is portrayed on social media and current trends in tourism reflecting apparent shifts in social acceptibilty. Over 200 travel companies and tour operators, for example, have committed to move away from selling or promoting venues offering elephant shows, rides, or other forms of direct contact between tourists and elephants [68]. Further research on other types of WTA in terms of how they are portrayed, and responded to, on social media in relation to current animal welfare guidelines (and, for threatened species, conservation assessments) is required to determine whether this is the case more broadly.

This is the first study of which we are aware that has attempted to assess the effect of Instagram’s wildlife selfie alert. There are few, if any, directly comparable initiatives on which to draw lessons. Although there is a body of research on ‘trigger warnings’ or ‘content warnings’ (e.g. [69–71]), these types of warnings are designed to warn viewers of potentially distressing content (i.e. to protect the viewer themselves), a fundamentally different objective to the selfie alert that aims to protect the animal in the image, other wild animals in a similar situation, and populations of the species shown. The use of counter-statements or warnings to prevent the spread of misinformation on social media is comparable to the wildlife selfie alert system in some respects (although the nature, and object, of harm associated with misinformation differs). One recent study in this area [72] was able to show that an unverified health-related tweet was less likely to be retweeted when it was accompanied by a counter-statement or warning. We were not able to monitor sharing of Instagram posts but we found no evidence that posts using a hashtag that triggered the warning were either less popular, or were perceived by commenters as more negative, than those that used hashtags that did not trigger the warning.

Our study had some limitations. Most relevant is that we were unable to assess how many people actually saw Instagram’s warning or, crucially, how many saw the warning and subsequently did not go on to view the associated posts. This has implications for the interpretation of post comments because our sample of comments will have been made either by users who were not shown the warning or by users who chose to ignore the warning. However, whilst this type of potential sampling bias might have influenced user response for those posts that used hashtags that triggered the warning (and could perhaps explain the apparent, albeit relatively minor, popularity effects observed), it does not negate our findings of rather limited, and inconsistent, triggering of the warning. Further, whilst the fact that the warning is only presented to users searching for hashtag terms has implications for our study design, it also represents a weakness in the implementation of Instagram’s warning. Indeed, posts that do not use any hashtags would neither trigger the warning or be detected in our initial search, highlighting further weaknesses in the system and also suggesting that our sample of posts might underestimate the prevalence of wildlife selfies on Instagram. In terms of study limitations, it is also possible that our failure to detect a statistically significant difference in sentiment scores between posts using hashtags that did and did not trigger the warning (for two of the pairs of hashtags) was due, in part, to limited sample size (number of comments and thus words available for analysis) and insufficient precision in estimated scores. There is little guidance on recommended sample size in sentiment analysis, but [73]’s analysis demonstrates increasing precision with increasing number of words up to 300 to 400 words, with little gain in precision for sample sizes over one to two thousand words. In our study, the number of words used in the estimation of sentiment scores varied between 217 and > 2,500 but was < 300 for only one of the six hashtags, so we tentatively conclude that sample size was sufficient. There are also interpretation issues in assigning sentiment scores. One example in this case is the word ‘jealous’, which is scored as negative, but in this context presumably means that the viewer likes what they see and is jealous of the person in the photo. Similarly, the phrase “no riding elephants” or “you should not ride elephants” is given a sentiment score of 0, but is not, in this context, a neutral comment, rather it is a negative response to a photo of a person riding an elephant. The use, and intended meaning, of specific emojis may also vary with demographic, psychological, and/or cultural differences among users [74, 75], and may be used ironically [43]. These issues are rarely given explicit attention in sentiment analysis studies using social media data, but mean that average sentiment scores, and even overall polarity, may be inaccurate (e.g. [44, 76]). In this case, however, whilst the precise numerical sentiment scores reported should be interpreted with caution, it is the comparison between pairs of hashtags, rather than the scores themselves, that is relevant to the questions posed in this study. Finally, whilst our sample of hashtags was not randomly sampled, and we cannot strictly extrapolate our findings beyond the particular hashtags used in this study, consistency in average post popularity and comment sentiment scores observed *between* pairs of terms (i.e. between #elephantselfie/s and #elephantride/s, Figs 4 and 5) together with the range of terms used (selfie, ride, hugs/cuddles) suggest that our sample of hashtags is probably representative of other non-similar hashtags used.

## Conclusion

In conclusion, whilst Instagram’s wildlife selfie alert system is well-intentioned and has some value, it needs to be implemented more widely, consistently, and triggered at different points in the user process, to achieve its intended purpose. For elephants specifically, there is growing emphasis among operators on responsible tourism [66, 68, 77]; currently, content posted on Instagram does not reflect this. Inadequacies associated with Instagram’s current focus on specific hashtags suggest that alternative approaches, such as image recognition might be more effective. Image recognition is already used in detecting serious crime online [78, 79], and the technology is well developed for identifying species in camera trap images (e.g. [80]). The International Fund for Animal Welfare in collaboration with Baidu (a Chinese technology company specialising in internet services) recently launched an artificial intelligence (AI) -powered tool to identify images of endangered wildlife products traded online [81], and World Animal Protection used image recognition alongside key word searches and machine learning to train social listening algorithms to identify inappropriate wildlife selfies online [82]. AI technology is used by a number of social media platforms, including Instagram and Facebook (both owned by Meta), to detect and remove content that violates platform standards or guidelines (e.g. [83]); currently, however, although Instagram prohibits coordination of poaching of endangered species and the sale of any live animals, Instagram’s Community Guidelines make no mention of inappropriate wild animal content, or of wildlife selfies specifically [84]. Sponsored advertisements posted on social media might also be more effective in informing people of the potential hidden harm to wild animals involved in animal selfie opportunities for tourists and the photo prop industry. In terms of assessing the success of this or any other related initiative (c.f. [85]), Instagram might also consider a mechanism whereby users can provide feedback to the warning itself to provide a measure of exposure (how many people actually see and read it), interest, and perceived relevance, which would in turn inform refinements and/or further educational initiatives as part of a co-design approach [86]. Further detailed analysis of wildlife selfie posts on social media, and viewer response to them, is needed, but preliminary observations suggest that our findings, and suggestions, are relevant not only to elephants used in tourism but to many other species commonly involved in wildlife selfies. Overall, more needs to be done by the major social media platforms to ensure that content permitted on social media (across platforms) does not involve, or is not associated with, threat to wild populations, animal cruelty, or other types of interactions with wildlife that are deemed detrimental to their welfare or otherwise breach community guidelines. Instagram and other large social media platforms undoubtedly have considerable resources and technological expertise at their disposal. We urge them to use these wisely in order to play their part in promoting healthy, fair, ethical and sustainable interactions between wild animals and growing human populations.

## Supporting information

S1 Appendix Expanded Table 1 showing all hashtags that we were able to identify associated with elephant selfie posts on Instagram. Data shown are the post volume (the number of posts in which the hashtag was used), whether Instagram’s pop-up warning was triggered (Y = yes, N = no), and whether any of the posts had been posted (i.e. the hashtag had been used) since 2017 (since the warning had been introduced). Note that not all posts using each hashtag conformed to the definition of elephant selfie used in the study (or were relevant to the aims of the study) but hashtags were included if at least one post was relevant (see footnote).
(DOCX)

S2 Appendix Country locations for 440 elephant selfie posts on Instagram using the hashtags #elephantselfie, #elephantselfies, #elephantride, #elephantrides, #elephanthugs, #elephantcuddles. (DOCX)

S3 Appendix Popularity (number of likes) of Instagram posts using each of six hashtags, where the first of each pair of terms triggers Instagram’s warning and the second does not. IQR = inter-quartile range, RCV_Q_ = (quartile-based) robust coefficient of variation = [IQR/median]*0.75; HL-estimator is Hodges-Lehmann estimator (or the sample median of all cross-sample pairwise differences).
(DOCX)
