# Supplementary material for: Elephant ‘selfies’: Evaluating the effectiveness of Instagram’s warning of the potential negative impacts of photo opportunities with wild animals
Source: PLoS One. 2023 Apr 6;18(4):e0283858. doi: 10.1371/journal.pone.0283858 (PMC10079110; doi:10.1371/journal.pone.0283858)
Supplement: S1 Appendix — Data shown are the post volume (the number of posts in which the hashtag was used), whether Instagram’s pop-up warning was triggered (Y = yes, N = no), and whether any of the posts had been posted (i.e. the hashtag had been used) since 2017 (since the warning had been introduced). Note that not all posts using each hashtag conformed to the definition of elephant selfie used in the study (or were relevant to the aims of the study) but hashtags were included if at least one post was relevant (see footnote). (DOCX) [file pone.0283858.s001.docx]

**S1 Appendix. Expanded Table 1 showing all hashtags that we were able to identify associated with elephant selfie posts on Instagram.** Data shown are the post volume (the number of posts in which the hashtag was used), whether Instagram’s pop-up warning was triggered (Y=yes, N=no), and whether any of the posts had been posted (i.e. the hashtag had been used) since 2017 (since the warning had been introduced). Note that not all posts using each hashtag conformed to the definition of elephant selfie used in the study (or were relevant to the aims of the study) but hashtags were included if at least one post was relevant (see footnote).

| **Search term** | **Post volume** | **Warning triggered (Y/N)** | **Used in posts since 2017 (Y/N)** |
| --- | --- | --- | --- |
| #elephantselfie | 22269 | Y | Y |
| #elephantselfies | 705 | N | Y |
| #elephantride | 82601 | Y | Y |
| #elephantrides | 16922 | N | Y |
| #elephantriding | 29952 | Y | Y |
| #elephantsanctuary | 156169 | Y | Y |
| #elephantsanctuaries | 100+ | N | Y |
| #elephantsanctuariesarenotbetter | 1 | N | Y |
| #elephantsanctuariesinthailand | 2 | N | Y |
| #selfiewithelephant | 654 | N | Y |
| #elephantsanctuaryphuket | 5884 | N | Y |
| #elephantbath | 12752 | N | Y |
| #elephantsanctuarychiangmai | 9436 | N | Y |
| #elephantsanctuarythailand | 1538 | N | Y |
| #elephantselfie🐘 | 100+ | N | Y |
| #elephantselfie🐘📸 | 6 | N | Y |
| #elephantselfiefail | 1 | N | N |
| #elephantselfiesarethebestselfies | 3 | N | Y |
| #elephantselfiesunday | 2 | N | N |
| #elephantselfiee | 2 | N | N |
| #elephantselfietime | 1 | N | N |
| #elephantselfie2 | 1 | N | N |
| #elephantselfie2014 | 1 | N | N |
| #elephantselfiecrew | 2 | N | Y |
| #elephantselfiefirsttime | 1 | N | N |
| #elephantselfiefriday | 1 | N | N |
| #elephantselfieinthewild | 1 | N | N |
| #elephantselfie**😈** | 1 | N | N |
| #elephantselfiefees | 1 | N | Y |
| #elephantselfie2017 | 1 | N | N |
| #elephantride🐘 | 1000+ | N | Y |
| #elephantriders | 1000+ | N | Y |
| #elephantrider | 1000+ | N | Y |
| #elephantridethailand | <100 | N | Y |
| #elephantride🐘❤ | <100 | N | Y |
| #elephantridephuket | <100 | N | Y |
| #elephantrideinjaipur | <100 | N | Y |
| #elephantridejaipur | 100+ | N | Y |
| #elephantride✔ | 3 | N | N |
| #elephantridebali | <100 | N | Y |
| #elephantrides🐘🐘 | <100 | N | Y |
| #elephantrideselfie | <100 | N | Y |
| #elephantrider🐘 | 4 | N | N |
| #elephantrideinthejungle | 9 | N | Y |
| #elephantrides🐘 | 12 | N | Y |
| #elephantridethroughtheriver | 1 | N | N |
| #elephantrideexperience | 3 | N | Y |
| #elephantrideinthailand | 7 | N | N |
| #elephantride🐘😍 | 4 | N | N |
| #elephantride🐘🐘🐘 | 5 | N | Y |
| #elephantridefordays | 4 | N | N |
| #elephantrideing | 6 | N | Y |
| #elephantrideinbali | 8 | N | Y |
| #elephantridebangkok | 1 | N | Y |
| #elephantridesaren | 5 | N | N |
| #elephantridekrabisafari | 3 | N | N |
| #elephantrideamerfort | 9 | N | Y |
| #elephantridethroughthejungle | 3 | N | N |
| #elephantridetour | 5 | N | N |
| #elephantridekerala | 4 | N | Y |
| #elephantridesinthailand | 3 | N | Y |
| #elephantrideswithjaiden4 | 4 | N | N |
| #elephantridesrock | 1 | N | N |
| #elephantridesbali | 4 | N | Y |
| #elephantrideindia | 2 | N | Y |
| #elephantridesafari | 3 | N | Y |
| #elephantridetoamberfort | 2 | N | Y |
| #elephantrideswithstrangers | 2 | N | N |
| #elephantridesarethebestrides | 3 | N | N |
| #elephantridesareexpensive | 3 | N | Y |
| #elephantridesphuket | 1 | N | N |
| #elephantridessrilanka | 3 | N | Y |
| #elephantridesareawesome | 1 | N | N |
| #elephantridesarebumpy | 2 | N | N |
| #elephantridesforgoodluck | 2 | N | N |
| #elephantridesinthailandarenotfun | 2 | N | N |
| #elephantridesareneklevel | 1 | N | N |
| #elephantridesarethebestrevenge | 1 | N | N |
| #elephantriding🐘 | 1000+ | N | Y |
| #elephantridinginthailand | 100+ | N | Y |
| #éléphantriding | 2 | N | N |
| #elephantridingthailand | 12 | N | Y |
| #elephantridingtour | <100 | N | Y |
| #elephantridingbali | 19 | N | N |
| #elephantriding🐘[leaf emoji] | 5 | N | Y |
| #elephantridinginindia | 2 | N | N |
| #elephantriding🐘[flag emoji] | 2 | N | Y |
| #elephantridinglicence | 5 | N | Y |
| #elephantridingexperience | 3 | N | Y |
| #elephantridinginsrilanka | 3 | N | Y |
| #elephantridinginlaos | 1 | N | N |
| #elephantriding❤ | 1 | N | N |
| #elephantsanctuarybles | 1000+ | N | Y |
| #elephantsanctuarybali | 100+ | N | Y |
| #elephantsanctuaryhartbeespoortdam | 100+ | N | Y |
| #elephantsanctuarykrabi | 100+ | N | Y |
| #elephantsanctuarykohsamui | 100+ | N | Y |
| #elephantsanctuarypattaya | 100+ | N | Y |
| #elephantsanctuarysamui | 100+ | N | Y |
| #elephantsanctuarykualagandah | 100+ | N | Y |
| #elephantsanctuaryhazyview | 100 | N | Y |
| #elephantsanctuarysouthafrica | 100+ | N | Y |
| #elephantsanctuaryplettenbergbay | <100 | N | Y |
| #elephantanctuarythecrags | 10 | N | Y |
| #elephantsanctuarylaos | <100 | N | Y |
| #elephantsanctuary🐘 | 28 | N | Y |
| #elephantsanctuarykenya | 12 | N | Y |
| #elephantsanctuarycambodia | <100 | N | Y |
| #elephantsanctuaryvangvieng | <100 | N | Y |
| #elephantsanctuarysrilanka | <100 | N | Y |
| #elephantsanctuarymalaysia | <100 | N | Y |
| #elephantsanctuarynairobi | 7 | N | Y |
| #elephantsanctuaryjungle | 8 | N | Y |
| #elephantsanctuaryaonang | 7 | N | Y |
| #elephantsanctuaryphuket🐘💗 | 16 | N | Y |
| #elephantsanctuaryubud | 7 | N | Y |
| #elephantsanctuarypark | <100 | N | Y |
| #elephantsanctuarykathuphuket | 15 | N | Y |
| #elephantsanctuarykhaosok | 9 | N | Y |
| #elephantsanctuarycrags | 3 | N | Y |
| #elephantsanctuarycamp8 | 4 | N | Y |
| #elephantsanctuarybotswana | 9 | N | Y |
| #elephantsanctuarybangkok | 6 | N | Y |
| #elephantsanctuarythailandchiangmai | 2 | N | Y |
| #elephantsanctuarykohlanta | 3 | N | Y |
| #elephantsanctuarychiangmai🐘 | 8 | N | Y |
| #elephantsanctuarylao | 4 | N | Y |
| #elephantsanctuaryknysna | 5 | N | Y |
| #elephantsanctuarychiangrai | 18 | N | Y |
| #elephantsanctuaryindia | 13 | N | Y |
| #elephantsanctuaryjunglephuket | 8 | N | Y |
| #elephantsanctuaryadventure | 11 | N | Y |
| #elephantsanctuarypuket | 1 | N | Y |
| #elephantsanctuarychaingmai | 9 | N | Y |
| #elephantsanctuarycarepark | 4 | N | Y |
| #elephantsanctuaryharties | 7 | N | Y |
| #elephantsanctuaryofthailand | 12 | N | N |
| #elephantbathing | 5000+ | N | Y |
| #elephantbathtime | 500+ | N | Y |
| #elephantbaths | 100+ | N | Y |
| #elephantbathing🚿 | 100+ | N | Y |
| #elephantbathingtime | <100 | N | Y |
| #elephantbathingchaingmai | 20 | N | Y |
| #elephantbathing🐘 | 10 | N | Y |
| #elephantbathe | 21 | N | Y |
| #elephantbath | 8 | N | Y |
| #elephantbatheing | 2 | N | N |
| #elephantbath🚿 | 3 | N | Y |
| #elephantbath🐘🛁 | 2 | N | Y |
| #elephantbath😍 | 2 | N | Y |
| #elephantbathingtime🐘 | 3 | N | Y |
| #elephantbaths🐘💦 | 1 | N | Y |
| #elephantbathes | 1 | N | Y |
| #elephantbathphuket | 3 | N | Y |
| #elephantbathing💦 | 1 | N | Y |
| #elephantbathsarethebest | 1 | N | N |
| #elephantbaththailand | 3 | N | Y |
| #elephantbath❤❤❤ | 1 | N | Y |
| #elephantbathrides | 2 | N | N |
| #elephantbathingday | 1 | N | N |
| #elephantbath💦 | 1 | N | Y |
| #selfiewithelephants | 100+ | N | Y |
| #selfiewithelephant🐘 | 14 | N | Y |
| #selfiewithelephants🐘🐘 | 3 | N | NA |
| #selfiewithelephant🐘✌😄 | 2 | N | Y |
| #selfiewithelephant✌ | 1 | N | Y |
| #selfiewithelephantsarehard | 1 | N | Y |
| #selfiewithelephante | 1 | N | N |
| #selfiewithelephantu🐘 | 1 | N | Y |
| #elephantwashing | 1000+ | N | Y |
| #elephantwashingtime | 3 | N | Y |
| #bathingwithelephants | 1000+ | N | Y |
| #elephanttouching | 8 | N | Y |
| #elephanttouchingandfeeding | 1 | N | Y |
| #touchinganelephant | <100 | N | Y |
| #eleselfie | 500+ | N | Y |
| #elephanthugs | 1000+ | Y | Y |
| #elephanthugsandkisses | 100+ | N | Y |
| #elephanthugssanctuary | <100 | N | Y |
| #elephanthugsarethebesthugs | 3 | N | Y |
| #elephanthugsarethebest | 8 | N | Y |
| #elephanthugs🐘 | 4 | N | N |
| #elephanthugsnotdrugs | 2 | N | Y |
| #elephanthugsarethecoolest | 1 | N | N |
| #elephanthugsarethebesttoo | 1 | N | N |
| #elephantcuddles | 1000+ | N | Y |
| #elephantcuddlesarethebest | 4 | N | Y |
| #elephantcuddlesarethebestcuddles | 1 | N | Y |
| #elephantcuddlesarethebestkindofcuddles | 1 | N | Y |
| #feedingelephants | 5000+ | N | Y |
| #feedingelephants🐘 | 100+ | N | Y |
| #feedingelephantsinthailand | <100 | N | Y |
| #feedingelephantsbananas | 8 | N | Y |
| #feedingelephants🐘😍 | 6 | N | Y |
| #feedingelephants🐘🐘😍 | 2 | N | N |
| #feedingelephantsinbali | 3 | N | Y |
| #feedingelepants🐘[flag emoji] | 1 | N | N |
| #feedingelephantswithmyhusband | 1 | N | N |
| #feedingelephantsbaby | 1 | N | N |
| #feedingelephantsattheorphanage | 1 | N | N |
| #feedingelephants🐘🐘🐘 | 1 | N | N |
| #feedingelephantssugarcane | 1 | N | N |
| #feedingelephants🐘🍌 | 1 | N | Y |
| #feedingelephantsthailand | 1 | N | Y |
| #feedingelephants🍏🥕🍊🍐 | 1 | N | N |
| #feedingelephantsbananas🐘 | 1 | N | Y |
| #feedingelephantsisnteasy | 1 | N | Y |
| #elephantfeeding | 5000+ | N | Y |
| #elephantfeedingtime | 100+ | N | Y |
| #elephantfeeding🐘 | 100+ | N | Y |
| #elephantfeeding🍌 | 14 | N | Y |
| #elephantfeedingcamp | 7 | N | Y |
| #elephantfeedings | 2 | N | Y |
| #elephantfeeding🍌🌎 | 2 | N | N |
| #elephantfeedingprogram | 1 | N | Y |
| #elephantfeeding🐘🍉 | 2 | N | Y |
| #elephantfeeding🍌🍌 | 1 | N | Y |
| #elephantfeeding🐘🍌[flag emoji] | 1 | N | Y |
| #elephantfeedingbathing | 1 | N | N |
| #elephantfeedingelephant | 2 | N | Y |
| #elephantfeedingvideo | 1 | N | N |
| #elephantfeedingandbathing | 1 | N | Y |
| #elephantfeedingproject | 1 | N | N |
| #elephantfeedingthailand | 1 | N | N |
| #elephantfeeding🐘🐘🐘🐘 | 1 | N | Y |
| #elephantfeedingw | 1 | N | Y |
| #elephantfeed | 100+ | N | Y |
| #bathingelephant | 100+ | N | Y |
| #bathingelephants | 1000+ | N | Y |
| #bathingelephants🐘 | 5 | N | Y |
| #bathingelephantsintheriver | 6 | N | Y |
| #bathingelephants🐘❤ | 2 | N | Y |
| #bathingelephantsinthailand | 3 | N | Y |
| #bathingelephantsinmud | 1 | N | Y |
| #bathingelephantinchiangmai | 1 | N | Y |
| #bathingelephantsinnepal | 1 | N | Y |
| #bathingelephantsonthailand | 1 | N | Y |
| #washingelephant | 100+ | N | Y |
| #washingelephants | 1000+ | N | Y |
| #washingelephantsinriver | 15 | N | Y |
| #washingelephantsinthailand | 7 | N | Y |
| #washingelephants❤🙌 | 1 | N | Y |
| #washingelephant🐘 | 1 | N | Y |
| #washingelephantinwaterfalls | 1 | N | N |
| #washingelephantsisseriousbusiness | 1 | N | Y |
| #washingelephantsnotriding | 1 | N | Y |
| #washingelephantsinbali | 1 | N | Y |

The following hashtags were used exclusively in posts advocating against direct contact with elephants: #elephantridesarecruel, #elephantridingistorture, #elephantridingsucks, #elephantridingisnotcool, #elephantridingisabuse, #elephantridingiswrong, #elephantridingiscruel, #elephantrides❌.
