# Supplementary material for: Elephant ‘selfies’: Evaluating the effectiveness of Instagram’s warning of the potential negative impacts of photo opportunities with wild animals
Source: PLoS One. 2023 Apr 6;18(4):e0283858. doi: 10.1371/journal.pone.0283858 (PMC10079110; doi:10.1371/journal.pone.0283858)
Supplement: S2 Appendix — (DOCX) [file pone.0283858.s002.docx]

**S2 Appendix. Country locations for 440 elephant selfie posts on Instagram using the hashtags #elephantselfie, #elephantselfies, #elephantride, #elephantrides, #elephanthugs, #elephantcuddles.**

| **Country** | **#cuddles** | **#hugs** | **#ride** | **#rides** | **#selfie** | **#selfies** | **Total** |
| --- | --- | --- | --- | --- | --- | --- | --- |
| [Africa] |  |  |  |  | 1 |  | 1 |
| Cambodia |  |  |  | 2 |  |  | 2 |
| China |  |  |  |  |  | 1 | 1 |
| India | 4 |  | 46 | 6 | 3 | 4 | 63 |
| Indonesia | 16 | 15 | 13 |  | 4 | 4 | 52 |
| Kenya | 1 |  |  | 1 |  |  | 2 |
| Laos |  | 1 | 3 |  |  |  | 4 |
| Malaysia |  |  |  |  |  | 1 | 1 |
| Myanmar |  |  |  |  | 1 |  | 1 |
| Nepal | 1 |  | 5 |  |  |  | 6 |
| South Africa | 6 | 18 | 2 |  | 5 | 2 | 33 |
| Sri Lanka | 4 |  | 6 |  |  |  | 10 |
| Thailand | 54 | 59 | 41 | 4 | 29 | 33 | 220 |
| Unknown | 9 | 4 | 5 | 2 | 14 | 3 | 36 |
| USA |  |  | 1 |  |  | 1 | 2 |
| Vietnam |  |  | 1 |  | 1 |  | 2 |
| Zambia |  |  | 1 | 1 |  |  | 2 |
| Zimbabwe | 1 |  |  |  |  |  | 1 |
| **Total** | **96** | **97** | **124** | **16** | **58** | **49** | **440** |
