# Supplementary material for: Elephant ‘selfies’: Evaluating the effectiveness of Instagram’s warning of the potential negative impacts of photo opportunities with wild animals
Source: PLoS One. 2023 Apr 6;18(4):e0283858. doi: 10.1371/journal.pone.0283858 (PMC10079110; doi:10.1371/journal.pone.0283858)
Supplement: S3 Appendix — IQR = inter-quartile range, RCVQ = (quartile-based) robust coefficient of variation = [IQR/median]*0.75; HL-estimator is Hodges-Lehmann estimator (or the sample median of all cross-sample pairwise differences). (DOCX) [file pone.0283858.s003.docx]

**S3 Appendix. Popularity (number of likes) of Instagram posts using each of six hashtags, where the first of each pair of terms triggers Instagram’s warning and the second does not.** IQR = inter-quartile range, RCV_Q_ = (quartile-based) robust coefficient of variation = [IQR/median]*0.75; HL-estimator is Hodges-Lehmann estimator (or the sample median of all cross-sample pairwise differences)

| # | No. posts | Min. | Median | IQR | RCV_Q_ | Max. | Mann-Whitney U test | HL-estimator (95% CI) |
| --- | --- | --- | --- | --- | --- | --- | --- | --- |
| #elephantselfie! | 57 | 101 | 178 | 152 | 0.64 | 24,367 |  |  |
| #elephantselfies | 48 | 10 | 54 | 63.5 | 0.88 | 590 | W = 2464.5  p < 0.001 | 112 (91, 148) |
| #elephantride! | 122 | 101 | 194.5 | 224.3 | 0.86 | 5,630 |  |  |
| #elephantrides | 16 | 3 | 67 | 173.5 | 1.94 | 5,364 | W = 1423.5  p = 0.003 | 105 (56, 165) |
| #elephanthugs! | 97 | 10 | 99.5 | 109.8 | 0.91 | 1,295 |  |  |
| #elephantcuddles | 96 | 3 | 51.5 | 66.3 | 0.97 | 9,068 | W = 6311  p < 0.001 | 39 (22, 57) |
